# Surgical Management of Adrenocortical Carcinoma: A Literature Review

**DOI:** 10.3390/jcm11195754

**Published:** 2022-09-28

**Authors:** Leonardo Rossi, Chiara Becucci, Carlo Enrico Ambrosini, Marco Puccini, Malince Chicas Vasquez, Benard Gjeloshi, Gabriele Materazzi

**Affiliations:** Department of Surgical, Medical and Molecular Pathology and Critical Area, University of Pisa, 56126 Pisa, Italy

**Keywords:** adrenocortical carcinoma, surgery, laparoscopic, minimally invasive, open surgery, oncological outcome

## Abstract

Background: Adrenocortical carcinoma (ACC) is a rare malignant tumor with a poor prognosis. Radical surgical resection with negative margins represents the only opportunity for a potential cure. This review provides a critical assessment of the existing studies regarding the surgical approaches for the treatment of ACC. Methods: This review was performed according to criteria reported in the Preferred Reporting Items for Systematic Reviews and Meta-Analysis (PRISMA) statement. The research was carried out using the PubMed electronic library. This review is limited to comparative studies evaluating minimally invasive adrenalectomy (MIA) and open adrenalectomy (OA) in adult patients affected by ACC. Results: A total of 14 studies were selected for the review, reporting that 2574 patients underwent adrenal surgery for ACC: 1779 (69.1%) by means of OA and 795 (30.8%) by means of MIA. Six studies considered OA to be superior to MIA, whereas eight studies reported that MIA is as effective as OA in highly selected cases. All studies were retrospective with a heterogenous selection of patients. Conclusions: Data regarding the management of MIA are scarce, heterogenous, and mainly based on retrospective studies. OA remains the gold standard approach for the management of ACC; however, MIA may play a role in selected cases treated in high volume institutions with experienced surgeons.

## 1. Introduction

Adrenocortical carcinoma (ACC) is a rare malignant tumor with an estimated incidence of 1–2 per million of individuals per year [1].

ACC is characterized by a poor prognosis with an overall 5-year survival rate ranging from 15% to 60%, depending on the stage at diagnosis [1,2]. Unfortunately, ACC is diagnosed as a metastatic disease for up to 20–40% of cases [2]. In patients with ACC confined to the adrenal gland or with a locally advanced disease, radical surgical resection with negative margins represents the only opportunity for a potential cure [3].

Initially described in 1992 by Gagner et al. [4], laparoscopic adrenalectomy (LA) has rapidly become the gold standard technique for benign adrenal tumors. Since then, other minimally invasive techniques were developed, such as robotic adrenalectomy (RA) and retroperitoneoscopic adrenalectomy, but their use was almost limited to benign tumors. On the other side, minimally invasive adrenalectomy (MIA) remains controversial for the treatment of ACC: indeed, few retrospective studies with conflicting conclusions handled this debated issue [3].

The rationale for this review was to maintain attention on this topic on which the literature is scarce and whose data are controversial. Many reviews include studies that take into account patients who have been operated on over long periods and do not include the most recent publications. This review provides a critical assessment of the existing research published in the last ten years regarding the surgical approaches for the treatment of ACC, focusing on the oncological efficacy of MIA and open adrenalectomy (OA).

## 2. Materials and Methods

This comprehensive review was performed according to criteria reported in the Preferred Reporting Items for Systematic Reviews and Meta-Analysis (PRISMA) statement [5]. The research was carried out using the PubMed electronic library; only studies written in English and published between 2012 and 2022 were included. The following keywords were used to perform the research: “laparoscopic adrenalectomy”, “minimally invasive adrenalectomy”, “laparoscopy”, “open adrenalectomy”, “adrenalectomy”, “adrenocortical carcinoma”, and “adrenal carcinoma”. This review is limited to comparative studies evaluating MIA and OA in adult patients affected by ACC without synchronous or bilateral lesions. Only articles with at least 8 patients per each surgical approach were included. Two independent authors (C.B. and L.R.) screened all papers retrieved by the aforementioned search strategy.

**Figure 1:**
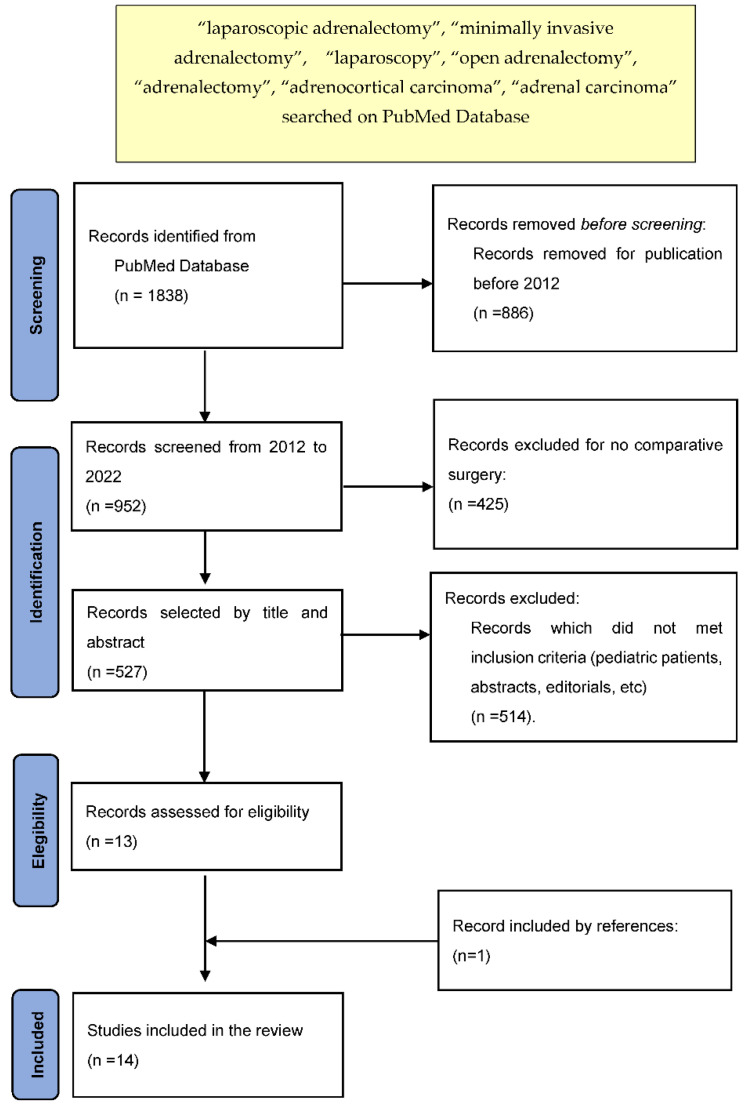
Prisma flow diagram.

Articles written by the same authors, editorials, abstracts, letters, comments, reviews, meta-analyses, and case reports were excluded as reported in the flowchart (Figure 1).

## 3. Results

A total of 14 studies were selected for the review. Overall, 2574 patients underwent adrenal surgery for ACC: 1779 (69.1%) underwent OA, whereas 795 (30.8%) underwent MIA. Of the 795 patients treated by MIA, 701 (88.2%) underwent LA, 12 (1.5%) underwent retroperitoneoscopic adrenalectomy, and 82 (10.3%) underwent RA.

Considering the ENSAT stage system, three studies included stage I-II patients [6,7,8]; six studies included stage I-III patients [9,10,11,12,13,14], and five studies included stage I-IV patients [15,16,17,18,19].

Features of the studies are summarized in Table 1 and Table 2. Overall, six studies confirmed OA as superior to MIA (Table 1), whereas eight studies reported that MIA is as effective as OA in selected cases, when oncological principles can be respected (Table 2).

## 4. Discussion

OA was routinely performed until the introduction of LA by Gagner in 1992 [4]. LA gained rapidly widespread acceptance and became the gold standard technique for benign functional and non-functional adrenal lesions. It is well-known that LA is associated with a lower complication rate, lower postoperative pain, lower blood loss, and shorter hospitalization and fasting period compared to OA [1,20,21]. Despite the abovementioned benefits, the role of LA in the treatment of ACC is still under discussion due to the lack of evidence to support its oncological safety [19].

Complete surgical resection remains critical for patients with ACC and offers the only opportunity for a cure. Indeed, adjuvant treatments are reported as highly ineffective, so the choice of surgical approach must be accurately assessed to allow patients to have the best chance of survival [12,19].

In 2012, Miller and colleagues [9] published a retrospective study on 156 patients with ACC (stage I-III): 110 treated with OA and 46 treated with LA. Despite larger tumors and a higher rate of stage III ACC, OA was associated with a lower rate of positive margins compared to LA, showing OA as superior in ensuring R0 resection (*p* = 0.04). Similarly, a higher rate of intraoperative capsular rupture (with increased risk of intraperitoneal tumor spread) was reported for the LA group compared to the OA group (30% vs. 16%, respectively). Moreover, the median overall survival (OS) of patients with stage II ACC was significantly longer in the OA group compared to the LA group (103 months vs. 51 months, respectively; *p* = 0.002) [9].

In addition, Miller et al. [9] reported that about 30% of patients preoperatively labelled as stage II were identified as stage III on postoperative histological examination, leading the authors to the crucial conclusion that pre- and intraoperative evaluation of ACC was not effective in detecting stage III ACC, limiting the potential role of MIA.

These outcomes were in accordance with other studies. Cooper et al. [16] reported in a large retrospective study on 302 patients with stages I-IV ACC (among them, 46 underwent LA) a longer OS, recurrence-free survival (RFS), and peritoneal-recurrence-free survival in patients who underwent OA compared to LA, after adjusting for pathologic T stage (*p* < 0.0001). Moreover, patients who developed peritoneal recurrence (statistically associated to LA) were less likely to undergo salvage surgery compared to other sites of recurrence [16].

Mir et al. [15] performed a study on 44 patients with ACC: 26 out of 44 underwent OA, whereas 18 out of 44 underwent LA. OA was associated with a 60% relative risk reduction in recurrence (hazard ratio 0.4) and a 50% relative risk reduction in mortality (hazard ratio 0.5), although these findings were not statistically significant. Notwithstanding, the two groups were not homogeneous in terms of tumor size, ENSAT stage, associated surgical procedures, and follow-up, limiting the power of the study. The authors concluded that patients with ACC should be considered for OA.

In 2016, Huynh et al. [12] published a large retrospective study on 423 patients with stages I-III ACC extracted from the National Cancer Data Base (NCDB); OA was performed in 286 cases, whereas MIA was performed in 137. On the basis of their results, the authors suggested caution in selecting MIA for surgical treatment of ACC. Indeed, a significantly higher rate of margin positivity in the MIA group compared to the OA group in patients with T3 tumors was documented (*p* = 0.0009); moreover, MIA was associated with a lower 3-year OS in stage II ACC patients (*p* = 0.04) [12].

In 2018, Wu et al. [8] published a retrospective analysis of patients operated on for localized ACC (stage I-II), dividing them into two groups on the basis of the approach: laparoscopic vs. open. Although the overall recurrence rate was the same between groups (52%), with no difference in terms of 5-year OS and RFS, the authors found that a combined pattern of recurrent disease (local and peritoneal) was greater in the laparoscopic group (42% vs. 22%, *p* = 0.035). Moreover, the mean time to local and peritoneal recurrence was significantly lower in the laparoscopic group (40 vs. 79 months, *p* = 0.048). The authors concluded that LA failed to achieve superimposable oncologic outcomes to OA in terms of the area and timing of the initial tumor recurrence, even for cases without extra-adrenal invasion and tumors smaller than 10 cm [8]. Similar findings were reported by Zheng et al. [13]. In this study, patients undergoing LA for ACC showed a significantly higher rate of local tumor bed recurrence as the first relapse site compared to OA patients (*p* = 0.03). Moreover, mean disease-free survival (DFS) was longer in the OA group (45 vs. 18 months, *p* = 0.023). Furthermore, the LA group showed a relative risk for recurrence of 2.1 compared to the OA group. Nonetheless, LA was associated with better perioperative data, in particular, shorter operative time (*p* = 0.004), lower bleeding loss (*p* = 0.001), and shorter postoperative hospital stay (*p* = 0.018) [13].

Notwithstanding, several authors suggested favorable oncological outcomes of MIA for ACC [6,7,14,17,18].

Lombardi et al. [6] published a multi-institutional study on 156 patients affected by ACC, 30 of whom were treated with MIA. The authors reported no statistically significant differences in terms of OS (*p* = 0.200), DFS (*p* = 0.120), recurrence rate (*p* = 0.497), and time to recurrence (*p* = 0.839), and claimed that MIA is a safe approach in localized ACC [6]. It is important to underline that the authors retrospectively excluded all patients with positive surgical margins after surgery. Moreover, although not statistically significant, some differences in terms of lymph node dissection were documented: indeed, only 1 patient (3%) underwent LND in the MIA group compared to 23 (18%) in the OA group (*p* = 0.079). This trend makes the two groups not perfectly homogeneous, limiting the generalizability of the results [6].

Fossa et al. [10] performed a single-center study on 32 patients with stage I–III ACC, 17 of whom were treated laparoscopically, whereas 15 were treated with the open approach. The authors reported better short-term and similar long-term outcomes in patients treated with the laparoscopic approach compared to those subjected to an open procedure: progression-free survival (*p* = 0.057), OS (*p* = 0.22), and recurrence rate (*p* = 0.33) were superimposable, whereas the grade of postoperative complication and the hospital stay was lower in the MIA group (*p* = 0.02 and <0.001, respectively). Nonetheless, MIA was also associated with smaller tumors (*p* = 0.002) with a trend toward the lower stage, both factors influencing the long-term prognosis [10]. Similarly, Donatini et al. [7] reported comparable oncological outcomes between LA and OA: in particular, no statistically significant differences were found in terms of disease-specific survival (*p* = 0.65), DFS (*p* = 0.96), OS (*p* = 0.634), and recurrence rate (*p* = 0.655), whereas a shorter hospital stay was correlated with LA (*p* < 0.02). The authors concluded that a strict patient selection is of primary importance to limit the risk of tumor capsule rupture and they reserve LA for lesions smaller than 10 cm to avoid tumor spillage [7]. Similar to Lombardi et al. [6], the authors excluded from their retrospective analysis all patients in which an R0 resection was not achieved, potentially introducing a selection bias [7].

Vanbrugghe et al. [11] performed a study on 25 patients operated on for ACC (16 laparoscopically, 9 open). Although the authors reported superimposable OS and RFS (*p* = 0.363 and *p* = 1.000, respectively), after adjusting for the WEISS score, LA was associated with an increased risk of recurrence and mortality (*p* = 0.004 and *p* = 0.018, respectively). Vanbrugghe et al. [11] concluded that the most important factor to obtain positive outcomes is an adequate surgical resection rather than the approach, and that LA may be appropriate in highly selected cases.

In 2016 Maurice et al. [17] performed an analysis of the quality of MIA for ACC using the NCDB. The authors reported that MIA was associated with a two-fold higher risk of positive surgical margin (*p* = 0.03), although, after adjusting for T stage, this difference remains significant only for T3 tumors. It is important to underline that approximately 25% of T3 tumors with a positive surgical margin in the MIA group was ≤6 cm (the traditional size cutoff for benign versus malignant disease). This may mean that these cases were preoperatively considered to be benign, and surgeons may not have adhered to the oncological principles of en bloc resection and wide local excision. Unfortunately, data on preoperative diagnosis were not available in the NCDB. This hypothesis may be supported even by the higher rate of LND for cN0 stage tumors associated with OA (19% vs. 2%, *p* < 0.01). The authors concluded that MIA affords comparable local control to OA for T1-2 disease, but inferior local control for T3 disease [17]. Afterwards, the same database was used by Calcatera et al. [19] to assess the surgical trends for the treatment of ACC. The authors documented that, in the period from 2010 to 2014, the use of MIA increased from 26% to 44%; moreover, RA increased from 5% to 16%. Additionally, the authors reported no differences between MIA and OA in terms of final margin status and OS. Nonetheless, it is noteworthy to underline that the open group was associated with a larger size and more advanced-stage tumors (*p* < 0.001 for both parameters) [19]. These results are in agreement with those reported by Lee et al. [18] in a multi-institutional study (with 13 tertiary care cancer centers involved). The authors concluded that MIA offers comparable surgical and oncologic outcomes to OA among highly selected patients with a tumor size less than or equal to 10 cm [18].

Finally, a recent study published by Kastelan et al. [14] reported no statistically significant differences in terms of RFS (*p* = 0.556) and OS (*p* = 0.767) between MIA and OA. Nevertheless, as previously reported in other papers, OA was performed in patients with larger tumors (*p* < 0.001) and a higher disease stage (*p* = 0.01) [14].

In the last two decades MIA has been increasingly used, even for the treatment of ACC [14,19]. Although initial studies raised criticism regarding the use of LA for the management ACC due to a higher risk of local recurrence and peritoneal carcinomatosis and lower OS [9,16,22], the progressive increase of the experience in minimally invasive surgery and the refinement of the techniques led to the reports of recent studies with comparable oncological outcomes between MIA and OA [7,10,14,18].

Recent systematic reviews and meta-analyses were performed on limited and heterogeneous data and led to conflicting conclusions on this debated issue [3,20,23,24,25]. The recently published guidelines on the management of ACC confirmed OA as the gold standard approach; however, they considered LA as acceptable in tumors smaller than 6 cm without any evidence of local invasion [26]. Moreover, some recent studies performed in specialized referral institutions reported that MIA is an appropriate approach even in tumors up to 10 cm if the oncologic principles are respected [7,14,18]. It is important to stress that the management of ACC should be limited to high-volume centers with surgeons with experience in these types of surgery.

This study harbors limitations which preclude the current authors from drawing definitive conclusions. Overall, the studies reviewed—most of them underpowered—are all retrospective and lack of randomization, introducing the risk of potential selection bias. Nonetheless, given the rarity of the disease and that localized ACC is often misdiagnosed and unknown to surgeons before operation, a prospective randomized study is not likely to be performed. Furthermore, some studies take into consideration partially overlapping cohorts of patients and draw different conclusions, making the analysis confusing and not definitive. Moreover, the studies analyzed include patients with heterogenous features, such as tumor size and different ENSAT stages, and who underwent different surgical operations (especially regarding en bloc resection or LND). Lastly, a lot of case series include patients operated on over long periods, often before 2010, with differences in terms of radiological accuracy for the diagnosis and surgical techniques, and in institutions with surgeons with different experience levels.

At our institution, we reserve LA for all lesions without extra-adrenal invasion regardless of the size of the tumor; nonetheless, all lesions bigger than 6 cm are handled considering the malignancy potential. Extra-adrenal extension is accurately preoperatively assessed with magnetic resonance imaging and/or computed tomography. In the case of intraoperatively suspecting local invasion, a prompt open conversion is performed.

## 5. Conclusions

ACC is a rare but aggressive endocrine tumor. Taking into consideration the unfavorable prognosis of the disease and the poor benefit of adjuvant therapy, a complete surgical resection adhering to oncological principles is mandatory in order to obtain a long-term cure.

OA remains the gold standard technique for the management of ACC. However, there is a trend toward an increased use of MIA which leads to endearing results when performed in selected cases in high volume institutions with experienced surgeons. Future larger studies may shed light on this controversial and debated issue.

## Figures and Tables

**Table 1:** Studies supporting open approach for the treatment of ACC.

Authors	Year of Publication	Patients, *n*	Surgical Approach, *n* (%)	ENSAT Stage	Tumor Size (Mean or Median), mm	Conversion, *n* (%)	R0, %	LND, *n* (%)	OS, Months or %	DFS, Months
Miller et al. [9]	2012	156	OA 110 (71%) LA 46 (29%)	I–III	OA 120 (range 50–280) MIA 74 (range 32–165)	-	OA 65 MIA 56	-	II: OA 103 MIA 51 III: OA 44 MIA 28	-
Mir et al. [15]	2013	44	OA 26 (59%) LA 18 (41%)	I–IV	OA 130 (range 58–218) MIA 70 (range 9–184)	5 (28%)	OA 61 MIA 61	OA 14 (54%) MIA 6 (33%)	(OA 54%) (MIA 58%)	OA 14 MIA 10
Cooper et al. [16]	2013	302	OA 256 (85%) LA 46 (15%)	I–IV	OA 120 (range 40–260) MIA 80 (range 10–150)	4 (9%)	OA 52 MIA 54	-	OA 110 MIA 54	OA 17 MIA 11
Huynh et al. [12]	2016	423	OA 286 (68%) LA 137 (32%)	I–III	OA 127 (SD ± 71) MIA 80 (SD ± 58)	-	OA 76 MIA 71	OA 88 (31%) MIA 4 (3%)	-	-
Wu et al. [8]	2018	44	OA 23 (52%) LA 11 (25%) RPSA 10 (23%)	I–II	OA 69 (SD ± 21) MIA 58 (SD ± 19)	1 (5%)	-	OA 3 (13%) MIA 0	OA 42 MIA 63	OA 22 MIA 25
Zheng et al. [13]	2018	42	OA 22 (52%) LA 20 (48%)	I–III	OA 101 (SD ± 36) MIA 63 (SD ± 22)	0	OA 100 MIA 100	-	-	OA 45 MIA 17

OA, open adrenalectomy; MIA, minimally invasive adrenalectomy; LA, laparoscopic adrenalectomy; RPSA, retroperitoneoscopic adrenalectomy; OS, overall survival; DFS, disease-free survival, LND, lymph node dissection; SD, standard deviation.

**Table 2:** Studies supporting minimally invasive approach for the treatment of ACC.

	Year of Publication	Patients, *n*	Surgical Approach *n* (%)	ENSAT Stage	Tumor Size (Mean or Median), mm	Conversion, *n* (%)	R0, %	LND, *n* (%)	OS, Months or %	DFS, Months or %
Lombardi et al. [6]	2012	156	OA 126 (81%) LA 29 (19%) RPSA 1 (1%)	I–II	OA 90 (SD ± 46) MIA 77 (SD ± 34)	0	OA 100 MIA 100	OA 23 (18%) MIA 1 (3%)	OA 60 MIA 108	OA 48 MIA 72
Fossa et al. [10]	2013	32	OA 15 (47%) LA 17 (53%)	I–III	OA 130 (range 60–140) MIA 80 (range 42–160)	2 (12%)	OA 80 MIA 71	-	OA 36 MIA 103	OA 8 MIA 15
Donatini et al. [7]	2014	34	OA 21 (62%) LA 13 (38%)	I–II	OA 68 (range 45–90) MIA 55 (range 22–80)	0	OA 100 MIA 100	-	(OA 81%) (MIA 85%)	OA 47 MIA 46
Vanbrugghe et al. [11]	2016	25	OA 9 (36%) LA 16 (64%)	I–III	OA 116 (range 12–200) MIA 62 (range 38–80)	0	OA 100 MIA 75	-	(OA 89%) (MIA 69%)	(OA 63%) (MIA 56%)
Maurice et al. [17]	2017	481	OA 320 (67%) LA 130 (27%) RA 31 (6%)	I–IV	OA 117 (IQR 85–160) MIA 75 (IQR 52–98)	24 (15%)	OA 83 MIA 80	OA 42 (13%) MIA 2 (1%)	(OA 62%) (MIA 58%)	-
Lee et al. [18]	2017	201	OA 154 (77%) LA 44 (22%) RPSA 1 (1%) RA 2 (1%)	I–IV	OA 109 (range 37–300) MIA 55 (range 30–160)	9 (19%)	OA 74 MIA 77	OA 63 (41%) 0	OA 54 MIA 91	OA 10 MIA 14
Calcatera et al. [19]	2018	588	OA 388 (66%) LA 151 (26%) RA 49 (8%)	I–IV	OA 124 (SD ± 69) MIA 89 (SD ± 62)	38 (19%)	OA 75 MIA 71	-	OA 55 MIA 53	-
Kastelan et al. [14]	2020	46	OA 23 (50%) LA 23 (50%)	I–III	OA 120 (range 70–250) MIA 75 (range 26–110)	0	OA 100 MIA 100	-	OA 149 MIA 109	OA 129 MIA 109

OA, open adrenalectomy; MIA, minimally invasive adrenalectomy; LA, laparoscopic adrenalectomy; RPSA, retroperitoneoscopic adrenalectomy; RA, robot-assisted adrenalectomy; OS, overall survival; DFS, disease-free survival, LND, lymph node dissection; SD, standard deviation; IQR, interquartile range.

## Data Availability

Not applicable.
